# Comparison of 2 Zero-Profile Implants in the Treatment of Single-Level Cervical Spondylotic Myelopathy: A Preliminary Clinical Study of Cervical Disc Arthroplasty versus Fusion

**DOI:** 10.1371/journal.pone.0159761

**Published:** 2016-07-21

**Authors:** Sheng Shi, Shuang Zheng, Xin-Feng Li, Li-Li Yang, Zu-De Liu, Wen Yuan

**Affiliations:** 1 Department of Orthopaedic Surgery, Renji Hospital, School of Medicine, Shanghai Jiaotong University, Shanghai 200127, P.R. China; 2 Department of Endocrinology, Renji Hospital, School of Medicine, Shanghai Jiaotong University, Shanghai 200127, P.R. China; 3 Department of Spine Surgery, Changzheng Hospital, Second Military Medical University, Shanghai, 200003, P.R. China; Universita degli Studi di Palermo, ITALY

## Abstract

**Objectives:**

Cervical disc arthroplasty (CDA) with Discover prosthesis or anterior cervical discectomy and fusion (ACDF) with Zero-P cage has been widely used in the treatment of cervical spondylotic myelopathy (CSM). However, little is known about the comparison of the 2 zero-profile implants in the treatment of single-level CSM. The aim was to compare the clinical outcomes and radiographic parameters of CDA with Discover prosthesis and ACDF with Zero-P cage for the treatment of single-level CSM.

**Methods:**

A total of 128 consecutive patients who underwent 1-level CDA with Discover prosthesis or ACDF with Zero-P cage for single-level CSM between September 2009 and December 2012 were included in this study. Clinical outcomes were evaluated using the Japanese Orthopaedic Association (JOA) score and Neck Disability Index (NDI). For radiographic assessment, the overall sagittal alignment (OSA), functional spinal unit (FSU) angle, and range of motion (ROM) at the index and adjacent levels were measured before and after surgery. Additionally, the complications were also recorded.

**Results:**

Both treatments significantly improved all clinical parameters (P < 0.05), without statistically relevant differences between the 2 groups. The OSA and FSU angle increased significantly in both groups (P <0.05). Compared with Zero-P group, ROMs at the index levels were well maintained in the Discover group (P < 0.05). However, there were no statistical differences in the ROMs of adjacent levels between the 2 groups (P > 0.05). Besides, no significant differences existed in dysphagia, subsidence, or adjacent disc degeneration between the 2 groups (P > 0.05). However, significant differences occurred in prosthesis migration in CDA group.

**Conclusions:**

The results of this study showed that clinical outcomes and radiographic parameters were satisfactory and comparable with the 2 techniques. However, more attention to prosthesis migration of artificial cervical disc should be paid in the postoperative early-term follow-up.

## Introduction

Anterior cervical discectomy and fusion (ACDF) is considered to be the standard surgical treatment for cervical spondylotic myelopathy (CSM) refractory to conservative treatment [1, 2]. As an effective selection of anterior reconstructions, plate-cage construct has been utilized popularly in the ACDF procedure. However, the plate-related complications such as adjacent level ossification and dysphagia are still of great concern [3, 4]. As a solution to these complications, a new zero-profile spacer (Zero-P, Synthes, Switzerland) for ACDF, constructed of a polyetheretherketone (PEEK) cage with integrated screws, has been developed and achieved satisfactory clinical results with fewer complications [5–9].

Compared with ACDF, cervical disc arthroplasty (CDA) has several theoretical advantages, including motion-preserving of index level and decreasing the stress of adjacent levels, and can be used as an alternative treatment to ACDF [10]. When the topic is clinically focused on the anterior reconstructions for single-level CSM, there is still no consensus on which technique is better between CDA and ACDF. The Discover artificial cervical disc with zero-profile (DePuy Spine, Raynham, MA, USA), a new prosthesis consisting of two endplates manufactured from titanium alloy and a polyethylene core, has been gradually used in the treatment of CSM. Besides, the efficacy and safety of Discover prosthesis has been confirmed by the previous studies [11–14]. However, previous study indicated that different elasticity modules of titanium and PEEK may also influence load sharing and stress distribution on the cervical kinematics [15], in addition to the different impacts of CDA and ACDF in the anterior reconstruction of cervical spine. Considering the aforementioned issues, it is necessary to compare the differences of the 2 new zero-profile implants in the treatment of 1-level CSM.

To our knowledge, there were no comparative studies on zero-profile artificial disc and anchored cage in the treatment of 1-level CSM. To compare the differences between zero-profile artificial disc and anchored cage in the treatment of 1-level CSM, we retrospectively analyzed the clinical outcomes, radiographic results, and complications of the patients with 1-level CSM who underwent CDA with Discover artificial disc or ACDF with Zero-P cage based on the 2-year follow-up.

## Materials and Methods

### Ethics statement

This study was approved by the institutional review board of Our Hospital (CZ-2015-N016). All subjects provided written informed consent. Research was conducted in accordance with the research principles in the Declaration of Helsinki.

### Patient demographics

Between September 2009 and December 2012, we performed CDA with Discover prosthesis or ACDF with Zero-P cage in 149 selected patients with 1-level CSM. A total of 128 consecutive patients with 1-level CSM who were treated by CDA with Discover prosthesis or ACDF with Zero-P cage who met the inclusion criteria were included in this study.

The inclusion criteria included: (1) symptoms of myelopathy, not responding to conservative treatment for more than 6 weeks; (2) objective evidence of 1-level CSM between C3 and C7; and (3) age range from 25 to 60 years. The exclusion criteria included several spondylosis, osteoporosis/osteopenia, cervical deformity, tumor, trauma, instability, metabolic bone disease, ossification of the posterior longitudinal ligament, congenital cervical stenosis, prior surgery of cervical spine, the disc height loss of index level > 50%, and a follow-up duration less than 24 months. All patients had the preoperative plain radiographs, computed tomography, and magnetic resonance imaging (MRI) of cervical spine.

These patients were observed for a follow-up of 2 years. All the patients were well informed about the potential disadvantages and expense of each construct and then chose which device they wanted to have implanted. 60 patients who received CDA with Discover disc prosthesis were included in Discover Group, and 68 patients who received ACDF with Zero-P spacer were included in Zero-P Group.

### Surgical procedure

After general anaesthesia, the patient was placed supine with mild neck extension. A right-sided transverse incision was implemented to expose the targeted segment. Then, the compressive materials were removed, including herniated disc, osteophytes and the posterior longitudinal ligament. After removal of cartilage endplate, the bony endplate was preserved to prevent device subsidence.

The Discover Group: the appropriate prosthesis was determined by both preoperative templating and intraoperative evaluation utilizing disc trials according to the protocol. After implant insertion, the fluoroscopy images were made to confirm whether or not the device is correctly located in the center of index disc space. Wearing a soft collar was not mandatory. The Zero-P Group: the appropriate cage was also determined by both preoperative templating and intraoperative evaluation utilizing trial cages. The PEEK cage packed with local excised bone and β-tricalcium phosphate was inserted into the disc space. Then, the screws were used cranially and caudally to fix the Zero-P cage. A soft collar was used for 4 weeks postoperatively.

### Data collection

The collected data included age, gender, operated level, operative time, blood loss, clinical conditions, and radiological parameters. Static and dynamic plain radiographs were obtained postoperatively at 2-day, 3-, 6-, and 12-month and 24-month (the final follow-up).

### Clinical evaluation

The clinical outcomes were evaluated using Japanese Orthopaedic Association (JOA) score and Neck Disability Index (NDI) functional score before surgery and postoperatively at each follow-up visit.

### Radiographic analysis

**Fig 1:**
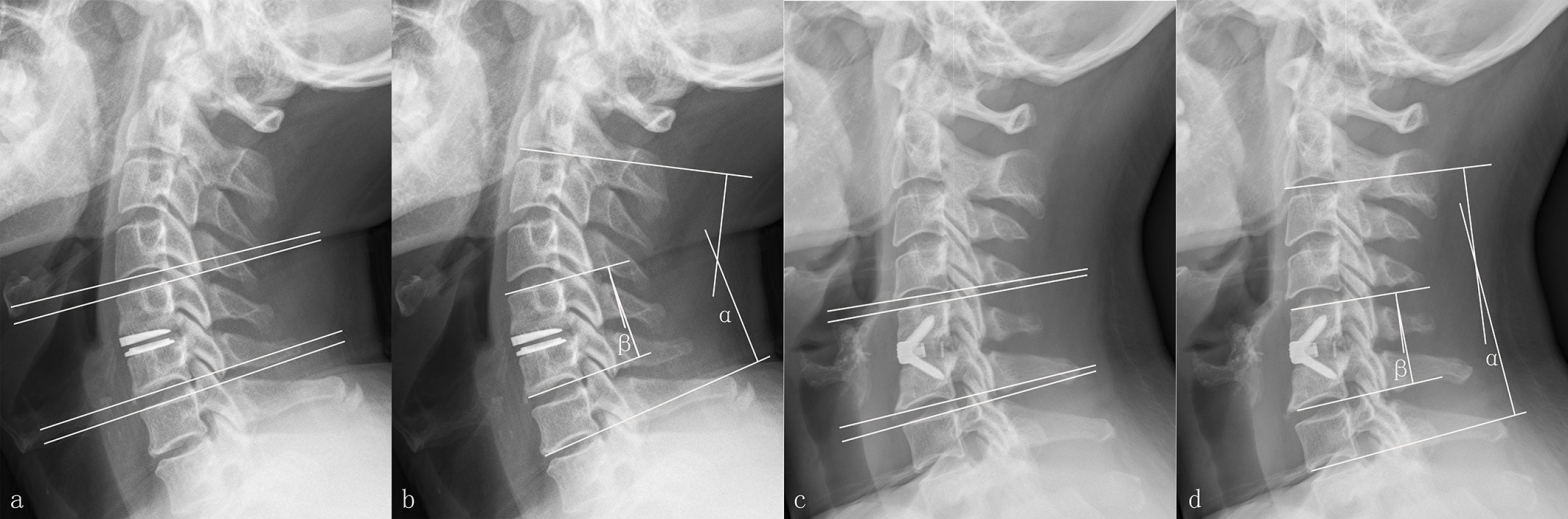
The methods about measurements of radiographic parameters. The range of motion (ROM) at the adjacent levels by disc space angle in the Discover group (a). The overall alignment of C2-C7 (α,OSA) and functional spinal unit (β,FSU) angle in the Discover group (b). The range of motion (ROM) at the adjacent levels by disc space angle in the Zero-P group (c). The overall alignment of C2-C7 (α,OSA) and functional spinal unit (β,FSU) angle in the Zero-P group (d).

Radiographic assessment included static and dynamic flexion-extension lateral radiographs in standing position. Radiological measurements were completed using the Picture Archiving and Communication System imaging system. The overall sagittal alignment (OSA) of the cervical spine was calculated by the Cobb angle between the inferior margins of C2 and C7 vertebral bodies on the lateral radiograph (Fig 1). The functional spinal unit (FSU) angle was defined as the angle between the superior margin of the cephalad vertebra involved and the inferior margin of the caudal vertebra involved. A negative value meant a kyphotic alignment. The range of motion (ROM) at the index and adjacent levels were assessed utilizing the FSU angle and disc space angle, respectively(Fig 1). The disc space angle was determined as the angle between straight lines drawn through the natural endplates. The sagittal ROM of the index and adjacent levels were measured using the flexion-extension lateral images. It should be noted that there were some patients’ images whose inferior endplates of C7 (2 patients in Discover group and 3 patients in Zero-P group) and the disc space angle of C7/T1 (9 patients in Discover group and 11 patients in Zero-P group) could not been seen, so we presented the statistics of OSA, FSU angle, and corresponding ROM at index and adjacent levels from the remaining patients. Solid fusion was defined as <2° on extension-flexion radiographs and the absence of a radiolucent gap between the endplate and the graft. To correct the intraobserver and interobserver reliability of the radiological measurements, two experienced observers, unrelated to the surgical procedures, independently assessed the radiographs of the patients. Each observer took the measurements twice for each radiograph, and mean values were used for statistical analysis. The occurrence of heterotopic ossification (HO) and migration were observed by the aforementioned observers in the follow-up. The performance of HO was graded on dynamic lateral radiographs according to McAfee’s criterion[16]. The radiographic findings of adjacent segment degeneration (ASD) above or below the index level included: loss of disc height (>30%), new osteophyte formation or enlargement of existing osteophytes, new or increasing anterior longitudinal ligament calcification.

### Statistical analysis

All data were analyzed using the Statistical Package for the Social Sciences, version 17.0 (SPSS Inc., Chicago, IL, USA). Categorical and continuous variables were presented as counts with/without percentage and mean ± standard deviation. The continuous data were evaluated for normal distribution using Shapiro-Wilk tests. Independent-samples t test or paired t test was adopted for normally distributed data. If the data showed a skewed distribution, Mann-Whitney U test was taken for differences. The Pearson’s chi-square test and Fisher exact test were used for assessing categorical variables. P<0.05 was considered statistically significant.

## Results

### Perioperative parameter

Of the 149 preliminary subjects, 21 patients were excluded because of unable to complete follow-up and missing preoperative radiological data. Finally, a total of 128 patients who successfully followed for a minimum follow up of 2 years were included in the present study, which corresponded to a follow-up compliance of 85.9% (128/149). There were 57 men and 71 women with a mean age of 47.0 years (range 32–60 years). The demographics of the included patients are shown in Table 1. In terms of perioperative parameters, no significant differences were observed between the 2 groups in age, sex, operated level, and blood loss. The mean operative time was 79.2±8.2 minutes in the Discover group and 86.5±6.9 minutes in the Zero-P group (P<0.05).

**Table 1:** Demographic data of patients.

	Discover Group	Zero-P Group	P
Age (years)	46.5±6.8	47.4±7.0	0.466
Gender (male/female)	24/36	33/35	0.333
Operated level			
C3-C4	5	5	0.975
C4-C5	20	21	
C5-C6	25	31	
C6-C7	10	11	
Smoker/non-smoker	9/51	7/61	0.422
With/without DM	3/57	4/64	1.000
Operative time (min)	79.2±8.2	86.5±6.9	0.000
Blood loss (ml)	78.4±14.2	77.1±14.5	0.625

DM, diabetes mellitus.

### Clinical assessment

For the 2 groups, the patients had significant neurological and functional activity improvements during the follow-up. Compared with preoperative scores, postoperative JOA scores significantly increased and NDI scores significantly decreased in the 2 groups (P<0.05; Table 2). However, there were no statistically significant differences for postoperative JOA scores and NDI scores between the 2 groups.

**Table 2:** Comparison of clinical outcomes between the 2 groups.

	Discover Group	Zero-P Group	P
JOA			
Preop	9.12±1.37	9.26±1.29	0.760
Postop 3-mo	14.33±1.30*	14.37±1.48*	0.864
Final FU	14.28±1.39*	14.24±1.26*	0.980
NDI			
Preop	20.03±2.80	19.90±2.09	0.633
Postop 3-mo	5.93±1.41*	6.00±1.36*	0.926
Final FU	5.77±1.24*	5.66±1.30*	0.638

JOA, Japanese Orthopaedic Association; NDI, Neck Disability Index; Preop, Preoperatively; Postop, Postoperative; FU, follow-up.

* P <0.05 compared with preoperative value.

### Radiographic analysis

#### Overall sagittal alignment and FSU angle

In the Discover group, the preoperative OSA and FSU angle were 5.97°±3.94°and 2.41°±2.53°, respectively (Table 3). The OSA and FSU angle at the final follow-up were 7.98°±2.92°and 3.71°±2.07°, respectively. Significant improvement in the OSA and FSU angle was achieved at the final follow-up for this group (P<0.05). In the Zero-P group, the OSA and FSU angle increased from 6.82°±3.08° to 8.36°±2.42°and from 3.12°±2.97°to 4.47°±2.28°at the final follow-up (P<0.05).

**Table 3:** Comparison of radiographic outcomes between the 2 groups.

	Discover Group	Zero-P Group	P
Overall sagittal alignment (°)			
Preop	5.97±3.94	6.82±3.08	0.187
Postop 3-mo	8.18±2.88*	8.72±2.56*	0.271
Final FU	7.98±2.92*	8.36±2.42*	0.395
FSU angle (°)			
Preop	2.41±2.53	3.12±2.97	0.202
Postop 3-mo	3.83±2.05*	4.60±2.42*	0.079
Final FU	3.71±2.07*	4.47±2.28*	0.057
ROM at index level (°)			
Preop	7.92±1.97	8.26±1.36	0.104
Postop 3-mo	8.09±1.82#	1.32±0.51*	0.000
Final FU	7.91±1.86#	1.03±0.32*	0.000
ROM at superior adjacent level (°)			
Preop	7.90±1.70	7.82±1.59	0.722
Postop 3-mo	8.12±1.48#	7.96±1.37#	0.557
Final FU	8.16±1.35#	7.76±1.37#	0.132
ROM at inferior adjacent level (°)			
Preop	7.84±1.57	7.74±1.54	0.723
Postop 3-mo	7.89±1.46#	7.94±1.53#	0.760
Final FU	8.04±1.49#	7.87±1.84#	0.758

Preop, preoperatively; Postop, postoperative; FU, follow-up; FSU. functional spinal unit; ROM. range of motion.

* P <0.05 compared with preoperative value.

# P >0.05 compared with preoperative value.

#### Range of motion at index level and adjacent levels

In the Discover group, ROMs of the index levels slightly decreased from 7.92°±1.97° preoperatively to 7.91°±1.86° at the final follow-up (P>0.05; Table 3). In the Zero-P group, ROMs of the index levels decreased from 8.26°±1.36° preoperatively to 1.03°±0.32° at the final follow-up (P<0.05). Compared with Zero-P group, ROMs of the index levels were relatively well preserved at each follow-up postoperatively in the Discover group. The fusion rate at postoperative 3 months and the final follow-up was 92.6% (63/68) and 100%, respectively. ROMs at the superior adjacent levels increased from 7.90°±1.70° to 8.16°±1.35° for Discover group and decreased from 7.82°±1.59° to 7.76°±1.37° for Zero-P group. Compared with preoperative ROMs, both groups showed insignificant difference of ROMs at the superior adjacent levels (P>0.05). ROMs of the inferior adjacent levels in the Discover group increased from 7.84°±1.76° to 8.04°±1.49°, but without significant difference (P>0.05). In the Zero-P group, ROMs of the inferior levels showed an insignificant increase at the follow-up compared with preoperative parameters (P>0.05). The difference between the 2 groups was found to be statistically insignificant (P>0.05).

#### Complications

**Fig 2:**
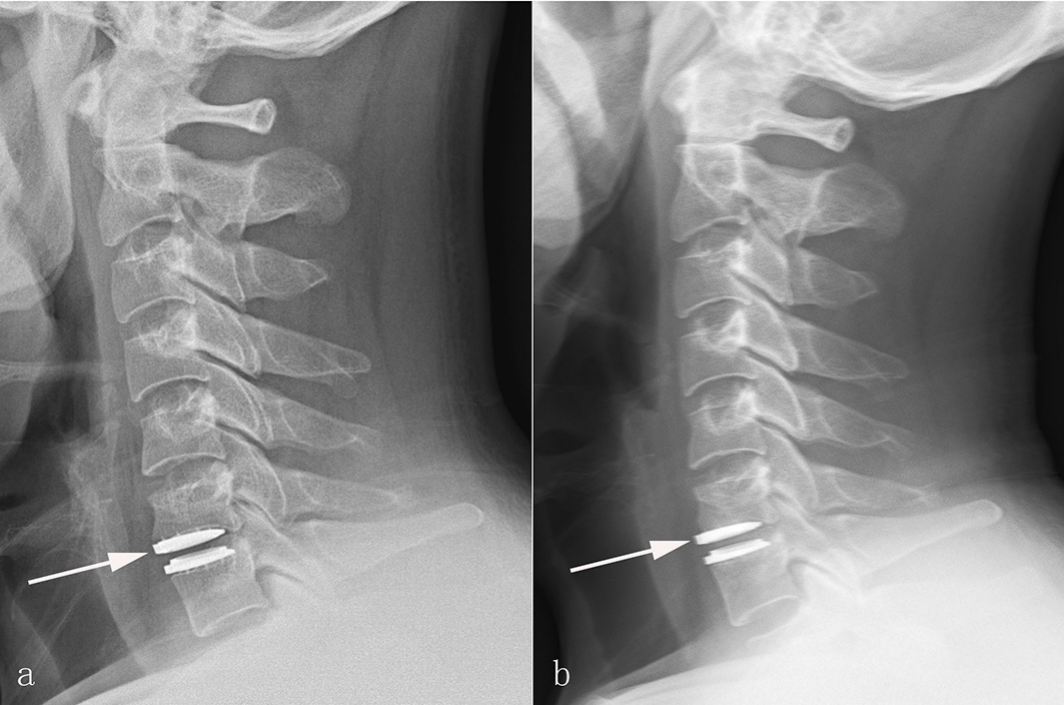
The representive case who suffered migration of Discover prothesis postoperatively. Postoperative 2-day (a), and final follow-up (b) lateral radiographs showing anterior migration of the superior endplate of Discover prosthesis (White arrows).

**Fig 3:**
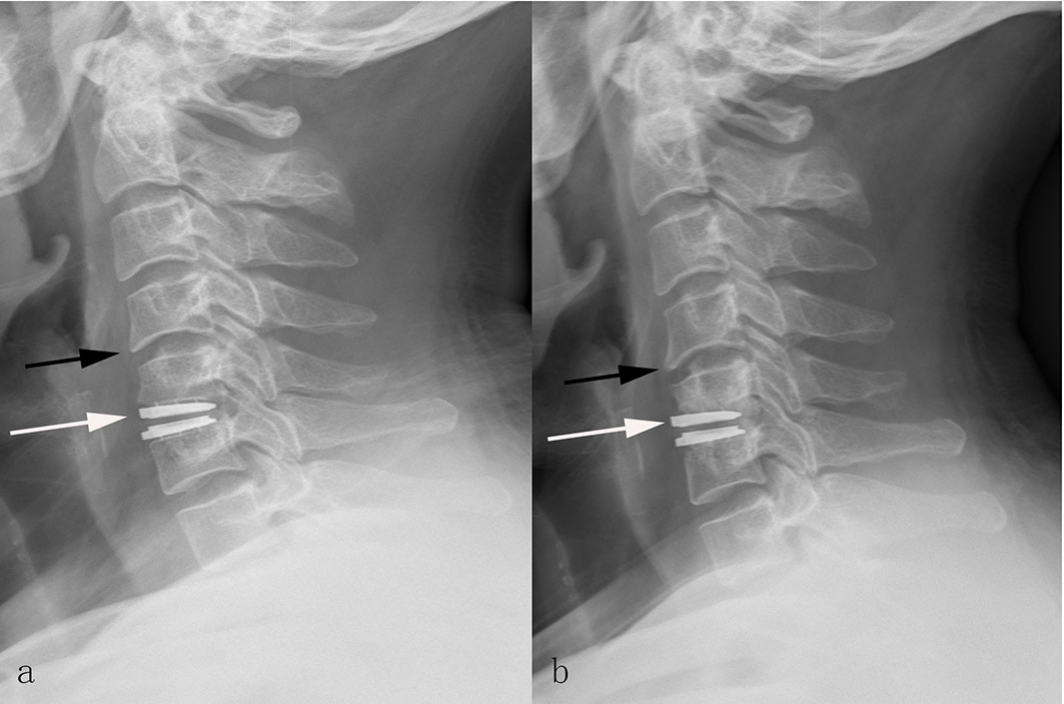
The representive case who suffered prosthesis subsidence and adjacent disc degeneration postoperatively. Postoperative 2-day (a), and final follow-up (b) lateral radiographs showing the superior endplate of Discover prosthesis subsiding into the cephalad vertebra and new anterior osteophyte formation in the patient after cervical disc arthroplasty with Discover prothesis. The white arrows indicate the subsidence of the prosthesis. The black arrows indicate the new anterior osteophyte formation.

**Fig 4:**
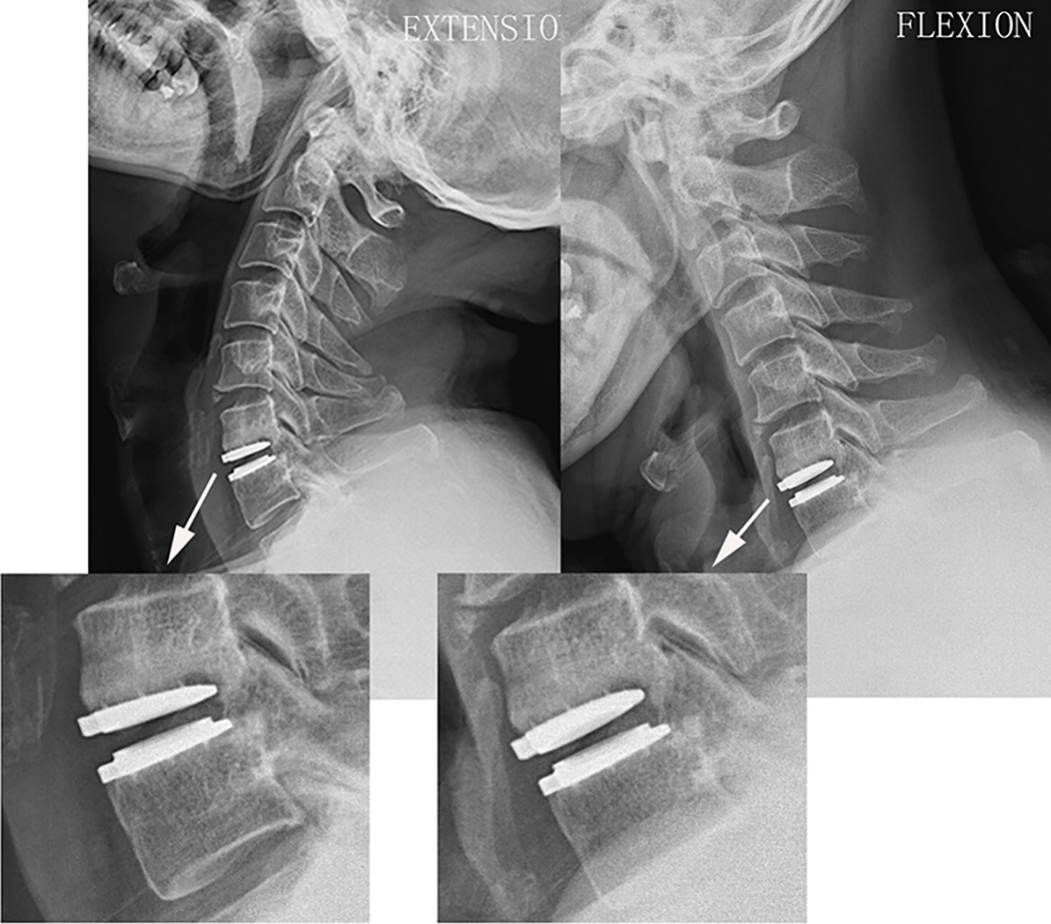
The representive case who suffered heterotopic ossification of Discover prosthesis postoperatively. Dynamic flexion-extension lateral radiographs at the final follow-up showing heterotopic ossification (Grade III) at the index level with significant restriction in the range of motion of Discover prothesis.

In the Discover group, dysphagia was detected in 6 patients (10.0%). Anterior migration of the Discover prosthesis was presented in 7 cases (11.7%; Fig 2). Of these 7 cases, two patients also suffered prosthesis subsidence and new anterior osteophyte at the superior adjacent level (3.3%; Fig 3). The prosthesis subsidence occurred at the anterior portion of the inferior endplate of cephalad vertebrae without significant reduction in the ROM. HO was found in 8 patients (13.3%; Fig 4). According to McAfee’s criterion, there were 4 Grade I cases, 3 Grade II case and 1 Grade III case. No patients suffered prosthesis expulsion, neurological deterioration or revision surgery. In the Zero-P group, dysphagia was presented in 7 patient (10.3%). Enlargement of existing osteophyte at the inferior adjacent level occurred in 2 patient (2.9%). Cage subsidence occurred in one patient (1.5%). Another patient suffered cerebrospinal fluid leak and recovered via conservative treatment (1.5%). The corresponding incidence of postoperative complications is shown in Table 4. No other complications were recorded in the 2 groups.

**Table 4:** Dysphagia, prothesis migration, subsidence and adjacent segment degeneration of patients between the 2 groups.

	Discover Group	Zero-P Group	P
Dysphagia	6/60(10.0%)	7/68 (10.3%)	0.956
Migration	10/60 (16.7%)	0/68 (0%)	0.000
Subsidence	2/60 (3.3%)	1/68 (1.5%)	0.600
Adjacent segment degeneration	2/60 (3.3%)	2/68 (2.9%)	1.000
Disc height loss	0/60 (0%)	0/68	1.000
New osteophyte formation or enlargement of existing osteophytes	2/60 (3.3%)	1/68 (1.5%)	1.000
New or increasing anterior longitudinal ligament calcification	0/60 (0%)	1/68 (1.5%)	1.000

## Discussion

ACDF has been reported to be the effective and safe procedure for the surgical treatment of cervical degenerative disc disorders since it was introduced to treat the degenerative diseases [17, 18]. For decades, the improvements of ACDF technique often have focused on increasing fusion rate, maintaining cervical lordosis and avoiding complications such as ASD [19–21]. Although the application of a plate-cage construct in ACDF has become a popular method of anterior reconstruction, it is also associated with plate-related complications such as dysphagia, and adjacent level ossification formation [22]. Meanwhile, rare complications and excellent outcomes have been reported in the application of Zero-P cage. Contrary to the conventional reconstruction, CDA is designed to preserve segmental motion and becoming popular as an alternative to ACDF [23–25]. Unfortunately, no protective effect of CDA on the kinematics of adjacent levels has been reported in the short- and mid-term follow-up[26–28]. Thus, it remains controversial whether arthroplasty accomplishes its major objective of preventing ASD in the long-term follow-up [29]. Considering the similar features of low-profile and the minimal effect on the adjacent segments, the comparison between Zero-P cage and CDA is of great interest.

Various factors contributing to unfavorable postoperative OSA have been studied, including overmilling of the endplates, preoperative sagittal alignment, positioning of the inserted device, design of the implant and intraoperative overdistraction[30]. Among these factors, some investigator reported the linear relationship between preoperative and postoperative overall cervical alignments [31]. However, this study showed a significant increase in the OSA of cervical spine after CDA or ACDF, which was consistent with the previous reports [32, 33]. Due to the built-in 7.0° lordotic angle of the Discover prosthesis, the FSU angle was corrected and maintained up to the final follow-up with no significant changes in FSU angle when compared with early postoperative performance. Similarly, the convex- or lordotic-shaped Zero-P cage was utilized to correct the FSU angle. The potential concerns of the 2 devices on achieving segmental lordosis may be related to the positive design. Moreover, it is well-known that the sagittal positioning of the implant in the treated level may play a key role in correcting the FSU angle. One reason for increased ROM at the early postoperative period is the removal of the posterior longitudinal ligament at the index level may increase the ROM. Another explanation may be related to the patients’ relief of preoperative symptoms after surgery and improved effort for dynamic flexion-extension radiographs [34]. Interestingly, no statistical differences occurred in the hypermobility of the adjacent segments between the 2 groups in this study. The superiority of CDA in the kinematics of adjacent segments in the 1-level CDA was not proven in this study, which was commensurate with the previous studies [26–28]. The possible reason may be the deviated center of rotation of prosthesis caused by migration may have a negative correlation with flexion-extension ROM [35], and affect the theoretically “natural” cervical kinematics. Furthermore, the adjacent segmental hypermobility caused by ACDF may not be presented via 2-yeay follow-up. As we known, the patients treated by CDA can have postoperative movement during the postoperative early-term follow-up [36, 37]. Nevertheless, the stability of artificial cervical disc is mainly reliant on the short fixed teeth in the postoperative early-term follow-up. Additionally, the prosthesis can hardly attached the irregular endplate, which will lead to a micro-gap between them. Additionally, Ren et al. [38] indicated that the C4/5 and C5/6 segments exhibited more complex kinematic characteristics in sagittal orientation in the view of Cobb angular velocity via dynamic extension-flexion images, which may result in the migration of implant. Thus, the maintenance of implant in excellent location during postoperative early-term follow-up may contribute to the protective effective on the kinematic of adjacent segments. The association between inadequate location of artificial cervical disc caused by prosthesis migration and the kinematics of adjacent segments, still requires further study.

The etiology of postoperative dysphagia may be multifactorial [39–43]. In this study, the 2 devices exhibited similar incidences of dysphagia. This finding also indicated that the zero-profile design of device may avoid stimulating the esophagus directly and reduce the incidence of dysphagia.

Although the reports regarding the anterior migration of Discover prosthesis were founded in the literature[12, 44], the reason of prosthesis migration has not been discussed. Thaler et al. [45] demonstrated that approximate 60% of Discover footprints did not match anatomic dimensions such as anteroposterior and mediolateral diameters. Thus, the prosthesis with a mismatched footprint cannot dissipate the axial load evenly, especially in the concentrated areas, which may induce the micromovement at the bone-implant interface, a possible reason for migration. Besides, the cervical endplates have the arched shapes in the sagittal plane, high in the center and low in the periphery. Chen et al. [46] also showed that the concavity apex was always located in the posterior half of the middle and lower cervical endplates, which resulted in the limited contact area and a micro-gap between the prosthesis and the endplate of the vertebrae postoperatively. Thus, the fixed teeth at the endplates of Discover prosthesis may provide insufficient resistance to the shear forces induced by the flexion-extension activities in the early postoperative duration. All the migrations occurring at early follow-up may explain the phenomenon in the study. Moreover, long-term fixation of the prosthesis was provided by both teeth and the osseointegration of the bone-implant surface. Thus, whether or not the complication might be avoided by the short-term wearing of cervical collar deserve further studies.

In view of prosthesis subsidence, it may be caused by inadequate bone quality, improper endplate preparation, unfavorable preoperative cervical alignment, or inappropriate load distribution associated with device design. It has been postulated that the posterior portions of cervical endplate are stronger than the anterior aspects and the middle of the endplate[47]. In this study, one prosthesis subsidence occurred at the anterior aspect, which was coincidently with the results of the aforementioned biomechanical study. Therefore, the proper size and adequate positioning of the prosthesis are important factors for preventing migration and subsidence. Moreover, the different elasticity modules of titanium and PEEK may influence load sharing and stress distribution in the anterior column of cervical spine, which also should be taken into consideration for the subsidence. As reported in other CDA clinical trials, the incidence of HO varied from 2.4% to 94.1% despite of the different types of the artificial disc prostheses[48]. Although the reason of HO remains unclear, it is certain that the incidence increases with time[49]. Yi et al. [50] indicated that a well-fitting prosthesis to endplate may prevent the occurrence of HO. Jin et al. [49] also proposed the importance of full coverage in preventing HO in the single-level CDA cases. Thus, it is necessary to make sufficient preoperative preparation for CDA.

There were several limitations in this study. First, the respective study prevented the randomization in design and had potential biases as well as confounding difficult to control. Second, the sample size in the present study was still not large enough and the follow-up was also not long enough to detect the potential difference, though neither dynamic hypermobility nor recurrence of neurological deterioration in the adjacent segments was detected in the study with a follow-up of 2 years. Third, only plain radiographs and flexion/extension lateral images were used to evaluate kinematics of adjacent segment, which may provide limited information due to the interference of osteophyte and lack of subtle differences from subjective MRI analysis. This limitation of study design results directly from the concern that MRI might be not available in all cases during the follow-up.

## Conclusions

The results of this study showed that clinical outcomes and radiographic parameters were satisfactory and comparable with the 2 techniques. Despite of the encouraging ROM at the index level, no significant differences between the 2 techniques were presented in OSA, FSU angle and ROMs at the adjacent levels. However, more attention to prosthesis migration of artificial cervical disc should be paid in the postoperative early-term follow-up.
